# Reducing Emergency Diagnostic Uncertainty with TRACE: Triage and Risk Assessment via Cost Estimation

**DOI:** 10.5811/westjem.50511

**Published:** 2026-02-27

**Authors:** Kian D. Samadian, Paul Chong, Boyu Peng, Ahmad Hassan, Kevin Shannon, Adriana Coleska, Abdel Badih el Ariss, Norawit Kijpaisalratana, Pedram Safari, Emma Chua, Daerin Hwang, Shuhan He

**Affiliations:** * Harvard Medical School, Massachusetts General Hospital, Department of Emergency Medicine, Boston, Massachusetts; † Walter Reed National Military Medical Center, Department of Ophthalmology, Bethesda, Maryland; ‡ Massachusetts General Institute of Health Professions, Boston, Massachusetts; § University of Virginia School of Medicine, Charlottesville, Virigina; || Pasadena City College, Pasadena, California; # Williams College, Williamstown, Massachusetts

## Abstract

**Introduction:**

Diagnostic uncertainty significantly impacts patient safety in emergency medicine, leading to missed diagnoses and severe harm. Current predictive models primarily emphasize diagnostic likelihood without explicitly addressing potential clinical harm from errors. We propose Triage and Risk Assessment via Cost Estimation (TRACE), a machine-learning framework that incorporates expected-value calculations, defined as the probability-weighted estimate of clinical harm, and patient similarity metrics to address both diagnostic accuracy and risk assessment.

**Methods:**

Using the Medical Information Mart for Intensive Care IV - Emergency Department dataset, we developed TRACE, comprising two modules: the expected value-powered triage index (TRACE-T), which calculates expected patient acuity from vital signs and chief complaints, and the patient similarity diagnosis engine (TRACE-Dx), which predicts diagnoses by identifying historically similar patients and weighing their outcomes by clinical harm. We assessed TRACE-T’s predictive performance, our primary outcome, using decision trees, random forests, and Lasso (least absolute shrinkage and selection operator) regression. The TRACE-Dx predictions, our secondary outcome, were evaluated through string matching (comparing diagnostic text) and sentence embedding similarity (comparing diagnostic phrases).

**Results:**

Our final analysis included a total of 2,501 patients from the dataset, due to requirements for diagnosis-string cleaning and computational demands of similarity calculations. Within this subset, TRACE-T significantly improved triage prediction accuracy, with the random forest classifier’s accuracy increasing from 0.605 to 0.705 (P = .04) and demonstrating a notable reduction in root mean square error from 0.635 to 0.541 (P < .001). The decision tree model improved from 0.467 to 0.593 (P = .78) but did not reach statistical significance. The TRACE-Dx generated five expected value-ranked predicted diagnoses per encounter (12,505 predictions across 2,501 patients) and achieved average sentence embedding and string match similarities of 93.3% (95% CI, 92.7–94.0%) and 92.5% (95% CI, 90.7–94.3%), respectively, indicating strong alignment with actual outcomes.

**Conclusion:**

Expected value-based clinical harm modeling with patient similarity scoring enhances triage accuracy and diagnostic prediction in emergency care. Triage and Risk Assessment via Cost Estimation provides interpretable, actionable insights that could be incorporated into real-time clinical workflows as decision-support tools to reduce diagnostic uncertainty and improve patient outcomes.

## INTRODUCTION

Diagnostic uncertainty remains a major challenge in acute care, where missed diagnoses can lead to serious or even fatal outcomes. Despite improvements in testing and imaging, about 5.7% of emergency department (ED) patients are misdiagnosed, and nearly 39% of serious harm involves critical conditions such as stroke, myocardial infarction, aortic aneurysm, spinal cord injury, or pulmonary embolism.^1^ These risks highlight the need for decision tools that account for both the likelihood of disease and the clinical consequences of missing it.

Machine learning (ML) and probabilistic approaches like Bayesian inference, fuzzy logic, and Monte Carlo simulations have been explored to manage uncertainty in complex clinical data.^2^–^5^ While effective at capturing intricate variable interactions, these models often fall short in translating predictions into clear, real-world clinical guidance. This gap is especially important in emergency medicine, where clinicians must distinguish between high-stakes conditions and more benign ones. Many current protocols still treat these presentations too uniformly.

Several risk-stratification models have been developed for acute care (eg, logistic regression, random forests with class weighting, and cost-sensitive classification methods), but these approaches often focus on improving classification accuracy rather than explicitly modeling the consequences of a misdiagnosis.^6^–^9^ Logistic regression provides probabilities but often requires manual thresholding to emphasize rare, high-risk outcomes. Random forests can adjust for class imbalance but may not reflect the true harm of missing a diagnosis such as stroke. Cost-sensitive methods adjust penalties for wrong classifications, but this often comes at the expense of transparency, which limits their use in frontline care.

Expected value analysis provides a more intuitive framework. By combining the probability of an outcome with the clinical harm associated with missing it or delaying treatment, expected value highlights which clinical risks matter most. Expected value is already widely used in finance, economics, and health policy, and has been applied in clinical areas such as cardiology, oncology, and medical decision-making.^10^–^12^ Incorporating expected value into predictive models could lead to more nuanced, risk-sensitive clinical guidelines, representing better how emergency physicians already think when weighing false positives against missed diagnoses.

Despite the promise of expected value-based models, few studies have systematically demonstrated how to integrate such an approach into a data-driven framework for guideline development. In the ED, clinicians face two core decision-making steps: first, estimating how urgently a patient needs care and, second, determining what the likely diagnosis is based on limited initial information. These steps (triage risk stratification and early differential diagnosis) require fast, high-stakes judgments that balance uncertainty, risk, and limited data. Both processes are fundamental to ED workflows and have direct implications for safety, resource use, and timely treatment. A single unified score can obscure the distinct cognitive tasks involved in triage vs diagnosis.

**Population Health Research Capsule**

What do we already know about this issue?

*Diagnostic uncertainty contributes to missed high-risk conditions in the emergency department (ED); most prediction models focus on probability rather than clinical harm*.

What was the research question?

*Can expected value and patient similarity modeling improve ED triage and early diagnostic prediction?*

What was the major finding of the study?

*Classification accuracy improved (0.61 to 0.71 [P = .04]), and regression error fell (0.64 to 0.54 [P < .001])*.

How does this improve population health?

*More accurate, interpretable triage and diagnosis support may reduce missed high-risk conditions, improve patient flow, and promote safer ED care at scale*.

To address this, we developed Triage and Risk Assessment via Cost Estimation (TRACE), a tool that helps clinicians make rapid, informed decisions by aligning with this two-step workflow. The TRACE tool comprises two distinct yet complementary modules:

1. TRACE-T: expected value-powered triage index
2. TRACE-Dx: patient similarity diagnosis engine.

By keeping triage and diagnosis separate, TRACE preserves interpretability while addressing both core clinical decisions. By demonstrating an expected value-driven approach in both domains, our framework aims to provide a proof of concept for more rigorous, data-grounded guidelines that address the realities of emergency medicine. Moreover, the black-box nature of many ML tools limits clinician trust and adoption, making interpretability a central requirement. Our objective in this study was to evaluate whether integrating expected value-based clinical harm modeling and patient similarity analytics improves early triage prediction and differential diagnosis generation in the ED.

## METHODS

### Study Design

This study was a retrospective data analysis using the publicly available, de-identified Medical Information Mart for Intensive Care IV - Emergency Department (MIMIC-IV-ED) database. The study did not include interaction with human subjects or identifiable patient data and was, therefore, exempt from institutional review board approval. All analyses were performed in accordance with established guidelines for retrospective chart review methodologies and with the Strengthening the Reporting of Observational Studies in Epidemiology reporting standards to ensure completeness and transparency.^13^,^14^

### Study Setting and Population

The MIMIC-IV-ED database was derived from ED encounters at the Beth Israel Deaconess Medical Center recorded between 2011–2019. We extracted patient vital signs, laboratory results, and chief complaints from electronic health records. For this study, we derived a curated subset of 2,501 encounters. This subset was required due to cleaning inconsistencies in diagnosis strings (from mixed *International Classification of Diseases*, 9^th^ and 10^th^ revisions [*ICD-9/10]*) formats, computational constraints for similarity calculations, and the need for high-quality inputs for the TRACE framework.

### Study Protocol

**Figure 1:**
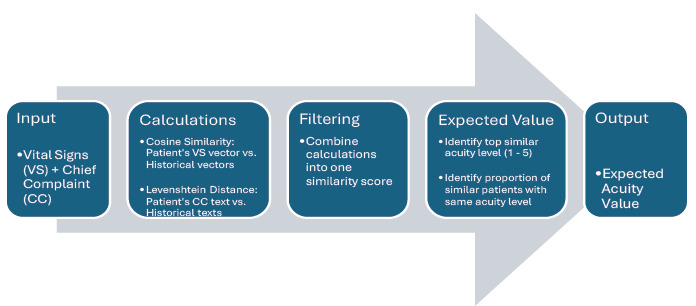
Depiction of the step-by-step process used by TRACE-T to generate an expected value-based acuity estimate based on a patient’s initial vital signs and chief complaint. Values indicate presentations with greater potential severity and higher triage priority. *TRACE-T*, Triage and Risk Assessment via Cost Estimation (triage prediction).

**Figure 2:**
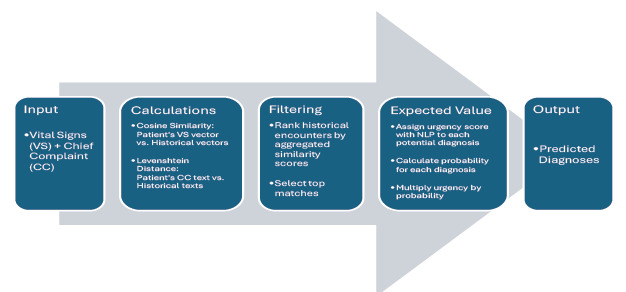
Depiction of how a patient-similarity diagnosis engine (TRACE-Dx) identifies the top five predicted diagnoses for a new patient based on the patient’s initial vital signs and chief complaint. *TRACE-Dx*, Triage and Risk Assessment via Cost Estimation (diagnosis prediction).

Diagnoses were drawn from both structured *ICD-9/10* codes and unstructured diagnosis strings. The TRACE framework included two primary modules: TRACE-T (triage prediction) and TRACE-Dx (diagnostic prediction). The TRACE-T module (Figure 1) uses patient vital signs and chief complaint to calculate expected acuity scores; TRACE-Dx (Figure 2) treats each patient as a new case and matches them to all remaining encounters in the analytic subset, using composite similarity metrics to generate potential diagnoses. The workflow was organized to reflect the stepwise clinical use of the system: 1) initial data pre-processing; 2) patient similarity matching; 3) expected value computation; 4) acuity prediction via TRACE-T; and 5) differential diagnoses via TRACE-Dx.

### Key Outcome Measures

Our primary and secondary outcomes were predictions of acuity level (TRACE-T) and diagnoses (TRACE-Dx), respectively, from presentation data. To calculate patient similarity, we applied two techniques: 1) cosine similarity to compare vital signs represented as numerical vectors; and 2) Levenshtein distance to compare chief complaint text. We normalized text and distances and averaged these two metrics to produce a composite similarity score. Each new patient was matched to historical cases using this score. Expected values were then calculated based on outcomes in the top-ranked similar cases using the formula, E(X) = ∑^n^_i=1_ x_i_ P(x_i_), where E(X) is the expected value, P(x_i_) represents the frequency of an outcome in the top matches and x_i_ represents either an urgency score for a diagnosis or a triage level.

### Data Analysis

For TRACE-T, we used the expected acuity value (either rounded or continuous) to predict ED triage levels. To accomplish this, we evaluated three model types commonly used in ML algorithms. Decision trees were included for interpretability because they create simple “if/then” decision pathways that resemble clinical reasoning. Random forests expand on this idea by generating many decision trees and averaging their results, which reduces the likelihood that any single tree leads to an unstable or misleading prediction. We used Lasso (least absolute shrinkage and selection operator) regression to estimate a continuous acuity score while automatically removing variables that did not meaningfully contribute to the prediction, which keeps the model focused and reduces overfitting. Model performance was assessed using accuracy, which reflects how often the predicted triage level matched the true level, and root mean square error (RMSE), which captures how far off the predictions were on average. We also calculated feature importance to determine which inputs contributed most strongly to each model’s output.

For TRACE-Dx, urgency scores for diagnoses were assigned using natural language processing of *ICD 9/10* descriptions. These scores provide an approximate sense of how serious a diagnosis may be based on its wording, rather than a precise measure of clinical severity. The model then identified each patient’s closest historical matches and selected the five diagnoses with the highest expected value. To evaluate how well these predicted diagnoses aligned with actual clinician-documented diagnoses (Table 1), we used two comparison techniques. The first was string matching, which measures overlap in the words used between the predicted and final diagnoses. The second was sentence-embedding similarity, which uses a pretrained language model to assess how close two diagnostic phrases are in meaning, even when the wording differs. These methods are well-established in clinical informatics research for evaluating diagnostic semantic similarity.^15^

To demonstrate how TRACE functions in practice, we provide a representative clinical example. A 54-year-old patient presents with chest pain and shortness of breath, and vital signs including a heart rate of 118 beats per minute and a respiratory rate of 24 respirations per minute. When processed through TRACE-T, the patient is matched to historical encounters that include presentations of both acute coronary syndrome and pulmonary embolism. Because these conditions carry a higher harm score if missed, the patient’s expected value-derived acuity score is elevated, and TRACE-T classifies the case as high acuity. The TRACE-Dx tool then uses the patient’s clinical presentation and similarity to prior encounters to generate five diagnoses ranked by expected value: pulmonary embolism; acute coronary syndrome; pericarditis; pneumonia; and anxiety-related chest pain. This illustrates how TRACE could support clinical decision-making at the time of presentation.

## RESULTS

**Figure 3:**
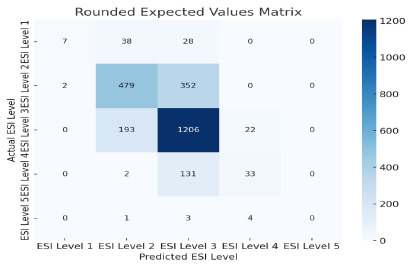
Prediction accuracy of TRACE-T using rounded expected values to estimate triage acuity levels. This confusion matrix compares predicted acuity levels (columns) with actual triage levels (rows). Darker cells represent correct predictions while lighter cells represent misclassifications. *ESI*, Emergency Severity Index; *TRACE-T*, Triage and Risk Assessment via Cost Estimation (triage prediction).

**Figure 4:**
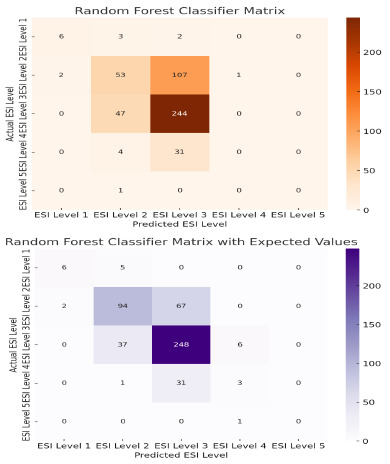
Performance of the random forest classifier with (4a) and without (4b) expected values in predicting triage acuity levels. Panels (a) and (b) display confusion matrices showing predicted vs actual acuity levels for models with and without expected value included. The greater value in the dark cell indicates improved accuracy and reduced spread of errors, highlighting the synergistic effect of expected value calculation in predicting acuity level of patients upon presentation. *ESI*, Emergency Severity Index.

In TRACE-T, the use of raw expected value scores alone yielded a classification accuracy of 0.690 (Figure 3). When incorporated into machine learning models, predictive performance improved substantially. The random forest classifier improved from 0.605 to 0.705 (*P* = .04), representing an increase in the predictive performance in the context of triage modeling (Figure 4), where even modest improvements can meaningfully affect patient flow and early risk identification. Lasso regression models showed the strongest gains, with raw expected value scores yielding an RMSE of 0.541 compared to 0.635 from the model alone (*P* < .001), indicating that it produced the most accurate continuous acuity estimates among the tested models. Because RMSE captures the average magnitude of prediction error, the lower value reflects better calibration and closer alignment with true triage levels.

**Figure 5:**
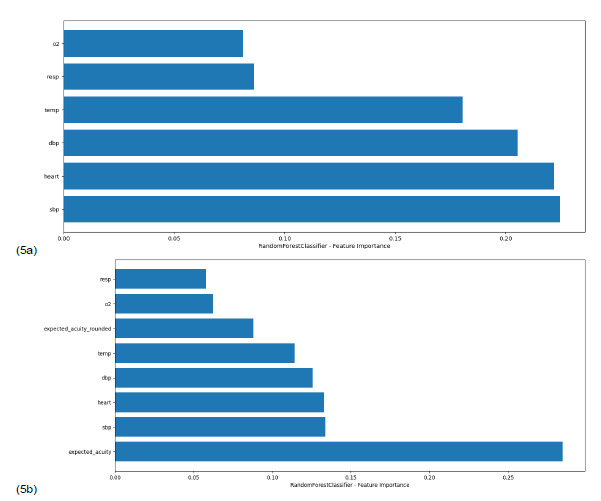
Feature importance analysis for the random forest classifier with (5a) and without (5b) expected values. These panels show the relative contribution of each input variable to the model’s prediction of acuity level. When expected value is included (a), it emerges as the dominant predictor, indicating that expected value captures critical information about patient severity that is not fully represented by individual vital signs. Without expected value (b), importance is distributed across several physiologic variables, but overall predictive performance is lower. This demonstrates the interpretability and predictive utility of integrating expected value into the triage model. Expected acuity rounded = rounded, whole-number calculated expected value for acuity level. Expected acuity = exact, raw calculated expected value for acuity level. *O_2_*, oxygen saturation; *resp*, respiration rate; *temp*, temperature; *DBP*, diastolic blood pressure; *heart*, heart rate; *SBP*, systolic blood pressure.

Although the decision tree classifier improved from 0.467 to 0.593 when expected value features were added, this result did not reach statistical significance (*P* = .78). This reflects limited predictive reliability, which is expected for binary decision models that are more prone to overfitting and lack the stabilizing benefit of ensemble averaging. Notably, in all models the raw expected value score consistently ranked as the most important feature, (Figure 5), demonstrating the relative importance of vital signs on triaging patients and underscoring its value in predicting acuity even within simplified or interpretable frameworks.

Following initial triage, clinicians must rapidly generate a working differential diagnosis, often with limited data. The TRACE-Dx tool is designed to augment their diagnostic reasoning by identifying the top potential diagnoses based on historical patient similarity and urgency. We analyzed 2,501 patient records and generated five expected value-predicted diagnoses per patient (yielding 12,505 total predictions). Among the top 1,000 results, string match similarity averaged 92.49% (SD 28.75%) and sentence embedding similarity averaged 93.32% (10.14%), indicating strong alignment between model predictions and actual clinical outcomes.

## DISCUSSION

This study introduces TRACE, a decision support framework that enhances both triage and diagnostic reasoning in emergency medicine. Through two distinct modules, TRACE-T and TRACE-Dx, we demonstrate how expected value calculations, combined with patient similarity metrics, can produce risk-adjusted, interpretable outputs aligned with clinical workflows.

The TRACE-T model targets one of the most critical decisions in emergency care: determining how urgently a patient needs evaluation. Using only vital signs and chief complaints, the system generates a continuous expected value score that reflects both the likelihood of serious illness and the clinical harm of missing it. When used in isolation, this score predicted triage levels more accurately than baseline models. When expected value was incorporated into machine-learning models, accuracy improved further across all classifiers.

Lasso regression models yielded the strongest performance improvements (*P* < .001), demonstrating the potential of expected value scores as dynamic, real-time indicators of patient acuity. The random forest model also showed significant gains (*P* = .04), reinforcing the value of combining expected value insights with more complex models, particularly useful in high-volume or high-variance ED settings. Although the decision tree model did not achieve statistical significance (*P* = .78), accuracy still improved from 0.467 to 0.593, suggesting a promising trend. More importantly, in all models, raw expected value consistently ranked as the top predictor, reinforcing its clinical utility, even in simpler, transparent models.

These improvements are not only statistically significant but also clinically meaningful. An approximate 10% gain in triage accuracy could lead to earlier identification of high-risk patients, more efficient resource use, and fewer delays for time-sensitive care. Feature importance analyses further support the value of expected-value modeling. Raw expected-value acuity consistently ranked as the most predictive variable, ahead of conventional inputs like heart rate or systolic blood pressure. Additionally, the raw expected value scores yielded a lower RMSE (0.541) compared to Lasso regression without expected value scores (0.635), indicating that even as a continuous variable, expected value captures nuance in triage prediction more effectively than standard modeling alone. Thus, raw expected value scores alone often matched or exceeded the predictive accuracy of certain machine-learning models.

Emergency physicians may, therefore, question the necessity of more complex modeling if a straightforward expected value approach already produces strong results. We suggest that expected value modeling and machine learning are complementary rather than competing. A simple expected value score (derived from just vital signs and chief complaint) can act as a fast, intuitive first-pass estimate that reflects how emergency physicians already think. Machine learning adds value by processing more variables (such as comorbidities or trends over time) to refine estimates when cases are less clear.^16^–^18^ This raises the question of whether more complex modeling is always necessary. In practice, expected value modeling and machine learning may be best viewed as complementary: simple expected value scoring provides an intuitive, rapid first-pass estimate of harm, while machine-learning architecture can integrate additional variables such as comorbidities or time-series data. This supports the core claim that expected value-augmented triage can enhance real-time risk stratification. By quantifying both the likelihood and potential consequences of serious illness, the expected value score translates complex patient data into a single, actionable estimate, aligned with how emergency physicians naturally prioritize care under diagnostic uncertainty.

Furthermore, the high similarity scores indicate that the TRACE-Dx model generates clinically plausible diagnostic impressions that align with clinician judgment These predictions were based only on the initial presentation, underscoring the strength of this expected value- and similarity-based approach. The model also showed low variability in similarity scores (SD 10.14%), suggesting consistent performance across diverse clinical cases. Prior work has validated the use of embedding and string-based methods in diagnostic prediction, and our findings support their continued use in acute care modeling. By highlighting high-risk, high-likelihood conditions early, TRACE-Dx may help reduce missed diagnoses especially in the case of time-sensitive conditions such as acute coronary syndrome or stroke or when senior support isn’t immediately available.

By maintaining separate models for triage and diagnostic reasoning, TRACE reflects the clinician’s actual workflow. Rather than collapsing both processes into a composite score, the modular design enables specific, actionable insights tailored to each decision point. Each module improved model performance with the addition of expected value scores and produced results that aligned with real-world decision-making. Importantly, our work builds on rapidly expanding literature demonstrating the utility of artificial intelligence in emergency care. Advanced machine learning and large language model-enabled systems have shown increasing promise in early identification of high-risk conditions, automated differential diagnosis generation, and real-time triage support.^19^,^20^ These tools illustrate the potential of artificial intelligence to augment clinician judgment in high-stakes settings. The TRACE tool contributes to this landscape by offering an interpretable, expected value-based approach that embeds clinical harm directly into predictive reasoning, differentiating it from probability-only or black-box systems. Taken together, our findings show that TRACE is a practical and effective framework for early decision support in emergency medicine.

The integration of expected value provides interpretable, risk-adjusted predictions that enhance accuracy without diminishing transparency. The TRACE tool could be incorporated into electronic health record systems via real-time triage widgets displaying expected value-based acuity scores, automated similarity-matching modules that refresh as new vitals or lab results arrive, and risk alerts embedded within existing ED tracking boards. For example, expected-value acuity scores could appear next to the triage nurse’s assignment of Emergency Severity Index, and TRACE-Dx differential suggestions could appear in the clinician’s initial assessment view as soon as chief complaint and vital signs are entered. These features would allow TRACE to enhance clinician judgment in real time. With further validation and integration into ED workflows, these tools could help reduce diagnostic error, improve patient flow, and support smarter, data-driven guideline development.

## LIMITATIONS

This study has several limitations. First, it relies on a single, retrospective dataset (MIMIC-IV-ED), which may have introduced bias and limits generalizability to other patient populations and healthcare settings. Furthermore, although this dataset includes over 420,000 encounters, our analysis consisted of 2,501 patients due to requirements for diagnosis-string cleaning and the computational demands of similarity calculations. Using optimized architectures or distributing computing would allow for a larger dataset, greater diagnostic diversity, and refinement of expected value-based urgency scoring. Additionally, validation in more diverse clinical environments and the inclusion of variables such as social determinants of health, will be essential in future work.

Specific limitations apply to the TRACE-Dx module. Although performance was strong, *ICD* code inconsistencies (eg, mixed *ICD*-9/10 formats, variable capitalization, and incomplete labels) limited our ability to reliably cluster or organize diagnoses into a hierarchical structure. Cleaner, standardized coding in future datasets could enable more advanced similarity modeling. Moreover, many records lacked clear primary diagnoses or included vague entries like “fever, unspecified” or “pain, other.” We removed non-specific terms to improve model validity, but this necessarily reduced diagnostic granularity and may have influenced overall similarity scores. Attempts to apply sentiment analysis to *ICD* titles for urgency scoring were also limited by the lack of domain-specific expertise in available sentiment models. Future work would benefit from datasets with explicit, consistently labeled diagnoses.

Implementing expected value-based tools like TRACE in real-time ED workflows will also require further study, including assessment of data availability, clinician training, and system integration. Future work will expand TRACE beyond triage and early diagnosis to include final diagnosis and treatment planning. By incorporating labs, imaging, and specialist input, we aim to test how initial expected value estimates align with final outcomes and to support more personalized care planning. This will help create a seamless continuum of care, spanning the entire patient journey from initial triage to definitive treatment.

## CONCLUSION

Expected value modeling, when integrated with patient similarity scoring, offers a powerful framework for early ED decision support. The TRACE system enhances both triage and diagnosis through interpretable, risk-adjusted modeling. By mirroring real clinical processes and supporting context-specific decision points, these capabilities could be incorporated into real-time clinical workflows as decision-support tools, offering clinicians triage guidance and diagnostic evaluation considerations informed by historical patient data to support evidence-based decision-making in emergency departments. Ultimately, TRACE may serve as the foundation for a new generation of data-driven, equitable emergency care systems.

## Figures and Tables

**Table 1:** Distribution of final diagnoses among 2,501 encounters included in a study of a machine-learning framework that incorporates expected-value calculations and patient similarity metrics to address both diagnostic accuracy and risk.

Diagnosis	Cases (%)
Hypertension	639 (25.5%)
Abdominal pain	401 (16%)
Chest pain	344 (13.8%)
Fracture	293 (11.7%)
Alcohol intoxication	244 (9.75%)
Heart failure	163 (6.52%)
Cellulitis	113 (4.53%)
Suicidal ideation	102 (4.07%)
Kidney failure	102 (4.07%)
Syncope	100 (4%)
